# Loss of PAFR prevents neuroinflammation and brain dysfunction after traumatic brain injury

**DOI:** 10.1038/srep40614

**Published:** 2017-01-17

**Authors:** Xiang-Jie Yin, Zhen-Yan Chen, Xiao-Na Zhu, Jin-Jia Hu

**Affiliations:** 1 Department of Anatomy, Histology and Embryology, Shanghai Jiao Tong University School of Medicine, Shanghai, 200025, China; 2 Department of Pharmacology, Shenyang Pharmaceutical University, Shenyang, 110016, China

## Abstract

Traumatic brain injury (TBI) is a principal cause of death and disability worldwide, which is a major public health problem. Death caused by TBI accounts for a third of all damage related illnesses, which 75% TBI occurred in low and middle income countries. With the increasing use of motor vehicles, the incidence of TBI has been at a high level. The abnormal brain functions of TBI patients often show the acute and long-term neurological dysfunction, which mainly associated with the pathological process of malignant brain edema and neuroinflammation in the brain. Owing to the neuroinflammation lasts for months or even years after TBI, which is a pivotal causative factor that give rise to neurodegenerative disease at late stage of TBI. Studies have shown that platelet activating factor (PAF) inducing inflammatory reaction after TBI could not be ignored. The morphological and behavioral abnormalities after TBI in wild type mice are rescued by general knockout of *PAFR* gene that neuroinflammation responses and cognitive ability are improved. Our results thus define a key inflammatory molecule PAF that participates in the neuroinflammation and helps bring about cerebral dysfunction during the TBI acute phase.

Traumatic brain injury (TBI) is characterized by a direct injury to the head which leads to a tissue damage followed by a secondary neuroinflammatory response^1^,^2^,^3^. TBI is a major cause of death and disability worldwide, resulting in large financial and social costs for the affected individuals as well as their families, especially in low- and middle-income countries^4^,^5^. The function of brain is abnormal in patients of TBI who show an acute and long-term neurological dysfunction, which is caused mainly by the pathological process including malignant brain edema and inflammatory response^6^,^7^. Although diagnosis and treatment methods are improving, the mortality rate associated with TBI has remained static and treatment is limited to palliative care^8^,^9^,^10^.

Inflammation, specially within the central nervous system after brain injury, which can cause secondary injury following the initial injury has been of extensive interest to researchers^11^,^12^,^13^. TBI has long been known to give rise to acute classical secondary neurogenic inflammation associated with inflammatory cytokine release^14^. To avoid this, many neuroprotective strategies have been developed to inhibit this process. However, the specific mechanisms associated with TBI related cytokine release are poorly understood^15^,^16^. Therefore a better understanding of the exact mechanisms involved in secondary injury after TBI are needed.

PAF is a potent central nervous system (CNS) phospholipid messenger, which is involved in platelet aggregation and mediated inflammatory responses. Furthermore, PAF has been reported to play an important role in many pathophysiological processes including cerebral edema and cerebral ischemia-reperfusion injury through interactions with PAFR^17^,^18^. PAFR, which belongs to G protein coupled receptors superfamily, is a seven transmembrane proteins that expressed extensively throughout the brain including microglia and neurons and has been reported to be activated by interating with PAF^19^,^20^. To determine the relationship between PAF and the inflammatory response after TBI, we explored development of inflammation in the brain of *PAFR* knockout in which the effects had on cognitive function.

In the present study, we found that TBI impaired the ability of learning and memory that a certain degree of protection was associated with platelet activating factor receptor knockout (*PAFR* KO). Mechanismly, we found deletion of *PAFR* could abolish the inflammatory response and neuronal apoptosis caused by TBI. Furthermore, blocking interactions between PAF and PAFR can protect neuronal spine structure and density as well as the integrity of the ultrastructure of brain tissue.

## Results

### Generation of *PAFR* knockout mice and biochemical validation

**Figure 1:**
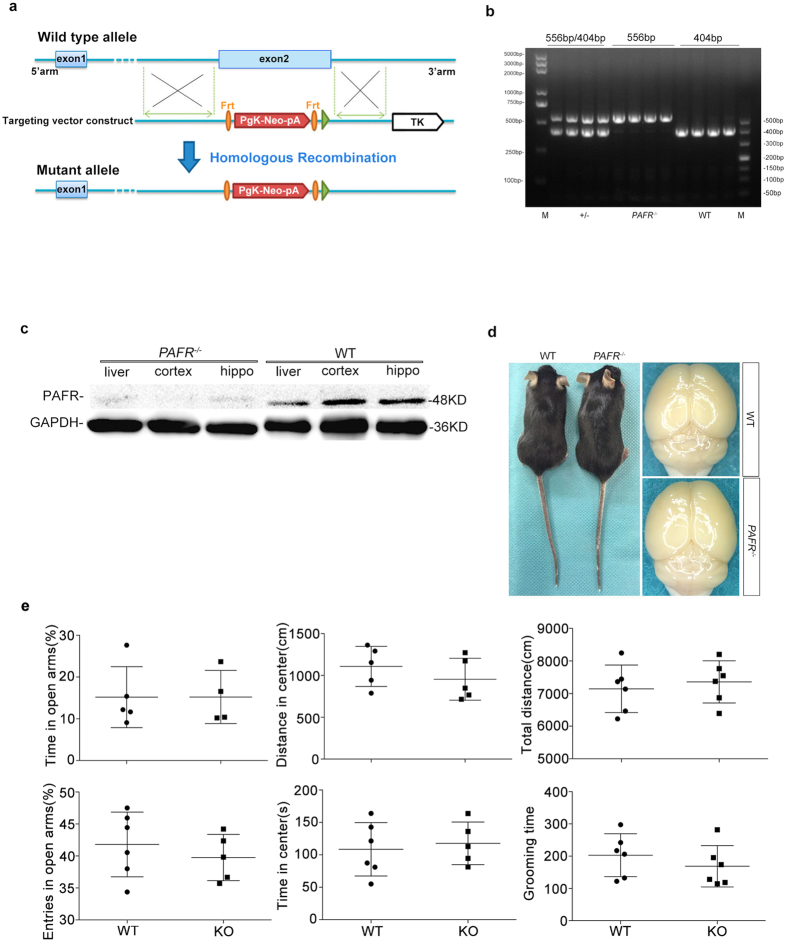
*PAFR* gene targeting strategy. (**a**) Exon 2 was replaced by the PgK – neo-pA, so that the gene transcription end with exon 1, transcription and translation is not complete and achieve the target gene knockout. (**b**) Genotypes of the mice were analyzed by PCR using DNA isolated from tail samples. The PCR product was 404 bp in WT mice and 556 bp in *PAFR* homozygous mice. *PAFR* heterozygous mice displayed both the 556 bp and 404 bp products. (**c**) PAFR protein expression in *PAFR*^−/−^ mice is significantly lower than WT mice. (**d**) There are no gross brain phenotypic differences between *PAFR*^−/−^ mice and WT mice. (**e**) *PAFR* mutant mice and wild type mice showed no difference in behaviors of EPM and OPT.

To identify the association of PAF with brain injury, we first got a PAFR protein null mutant in which the exon2 of *PAFR* gene was knock-out (Fig. 1a). The *PAFR* gene knock-out (*PAFR*^−/−^) was validated by PCR genotyping that wild-type (WT) mice showed a band of 404 bp while mutant was 556 bp (Fig. 1b). We further determined the protein level of PAFR with PAFR antibody from tissue of hippocampus, cortex and liver respectively and we found a dense band in WT mice while little in *PAFR*^−/−^ mice (Fig. 1c). The *PAFR*^−/−^ mice appeared to be healthy, fertile and long lived until adulthood. Brain of *PAFR*^−/−^ mice looked normal (Fig. 1d) and the mutant mice did not show different behaviors compared to WT mice. We further detected the natural behaviors of mice using open field and elevated plus maze tests, and found no difference in behaviors including fear, anxiety, athletic ability and life habits indicated by grooming time between two groups (Fig. 1e). These data indicate *PAFR* gene knock-out did not alter the innate physiology and behaviors of mice.

### Spatial learning ability and memory after TBI were improved in *PAFR* ^−/−^ mice

**Figure 2:**
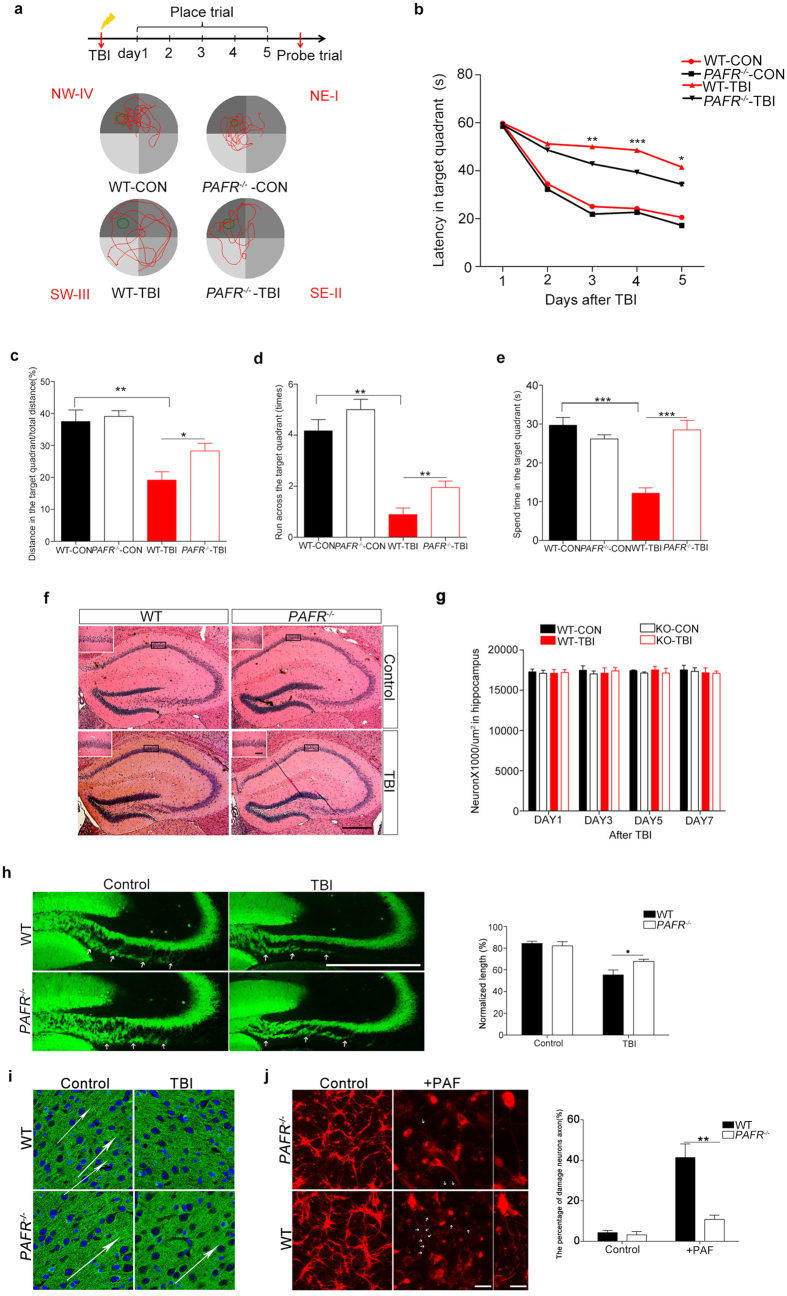
Effects of PAF-R KO on MWM performance in *PAFR*^−/−^ mice. (**a**) MWM experimental flow chart and representative images of the path chart of each experimental group in the MWM. WT mice spent significantly more time in the target quadrant trajectory than *PAFR*^−/−^ mice (n = 8). (**b**) The escape latency of the WT-TBI group was significantly longer than that of the *PAFR*^−/−^-TBI group. Both *PAFR*^−/−^-TBI and *PAFR*^−/−^-CON mice had significantly decreased escape latency times compared to their WT counterparts at day 3, 4 and 5, respectively. (**c**) Distances swam in the target quadrant in the probe trial were significantly shorter for WT-TBI mice than for *PAFR*^−/−^-TBI and WT-CON groups, respectively. (**d**) The number of platform crossings in the probe trail increased significantly in the *PAFR*^−/−^-TBI group compared with the WT-TBI group. (**e**) Signifciant differences were found among the *PAFR*^−/−^-TBI group and WT-TBI group in the time spent in the target quadrant during the probe trial. *P < 0.05, **P < 0.01, ***P < 0.001, one-way ANOVA. (**f**) Sections of hippocampal tissue from each experimental group stained with hematoxylin eosin stain showed no morphological differences between the groups. Scal bar, 500 μm. (**g**) Quantification of neurons at wild type and *PAFR*^−/−^ mice in hippocampus after TBI. n values of mice are indicated no difference. (**h,i**) Comparison of axonal length projected from DG of hippocampus and the direction of the axon in cortex between wild type and *PAFR*^−/−^ mice, the difference between WT-TBI and *PAFR*^−/−^-TBI mice is statistically significant. (n = 6) (**j**) Comparison of axonal fracture between wild type and *PAFR*^−/−^ primary neurons, the difference between WT and *PAFR*^−/−^ primary neurons after using PAF is statistically significant. *P < 0.05, **P < 0.01, ***P < 0.001. One-way ANOVA. Scale bars, 100 μm, 50 μm.

To determine how PAF mediates inflammatory response after brain injury, traumatic brain injury (TBI) was introduced. Inflammatory response caused by brain injury always led to an acute neurological dysfunction such as learning and memory defect^14^. Therefore we investigated the effect on spatial learning and memory ability with Morris water maze (MWM) after TBI. The performance in the probe trial of the MWM on day 6 was examined by analyzing the percentage of time spent swimming toward the platform (Fig. 2a). The shortest possible time to find the platform is interpreted as a better learning and memory^21^. We found no difference of spatial learning and memory between WT mice and *PAFR*^−/−^ mice in MWM task (Fig. 2a), however, the WT mice suffered from TBI spent longer time to found the target quadrant and showed lower accuracy compared to untreated WT mice and *PAFR*^−/−^ mice, indicating an imparied memory in WT mice after TBI (Fig. 2b). While the *PAFR*^−/−^ mice showed comparable behaviors including the ratio of distance, run across and spend time in the target quadrant whether TBI or not (Fig. 2c–e), which were better than WT-TBI group. These results suggest impaired cognitive function by TBI could be prevented by loss of *PAFR*, a protein contributes to inflammatory response caused by PAF.

In view of the correlation between hippocampus and spatial learning and memory, we asked whether TBI or absence of *PAFR* will damage hippocampus. Histological analysis by hematoxylin and eosin staining of the hippocampus revealed no obvious differences in either morphology or numbers of hippocampal neurons between WT mice and *PAFR*^−/−^ mice before or after TBI (Fig. 2f and g). We further observed the axonal change using immunostained with anti-calbindin that specifically labels the mossy fiber axons projected from DG neurons, and found a shorter length of infra-mossy fiber axons in WT-TBI mice compared to other groups (Fig. 2h). Moreover, the axons in cortex of WT mice or *PAFR*^−/−^ mice displayed an orientation that similar to the neuronal migration were disrupted after TBI in WT mice but not *PAFR*^−/−^ mice (Fig. 2i). To determine whether PAF directly played a harmful effect on axons, we cultured the primary neurons from pups of WT mice or *PAFR*^−/−^ mice and found a healthy develop of neurons with littel axonal fracture. However, 24 hr after using 5 μmol/L PAF the WT neurons displayed sharply increased axonal fracture which was suppressed by the deletion of *PAFR* (Fig. 2j). These data indicate that the impaired axon development after TBI may be caused by the PAF which could be prevented by loss of *PAFR*.

### Fewer glial cells were activated in *PAFR* ^−/−^ mice after TBI

**Figure 3:**
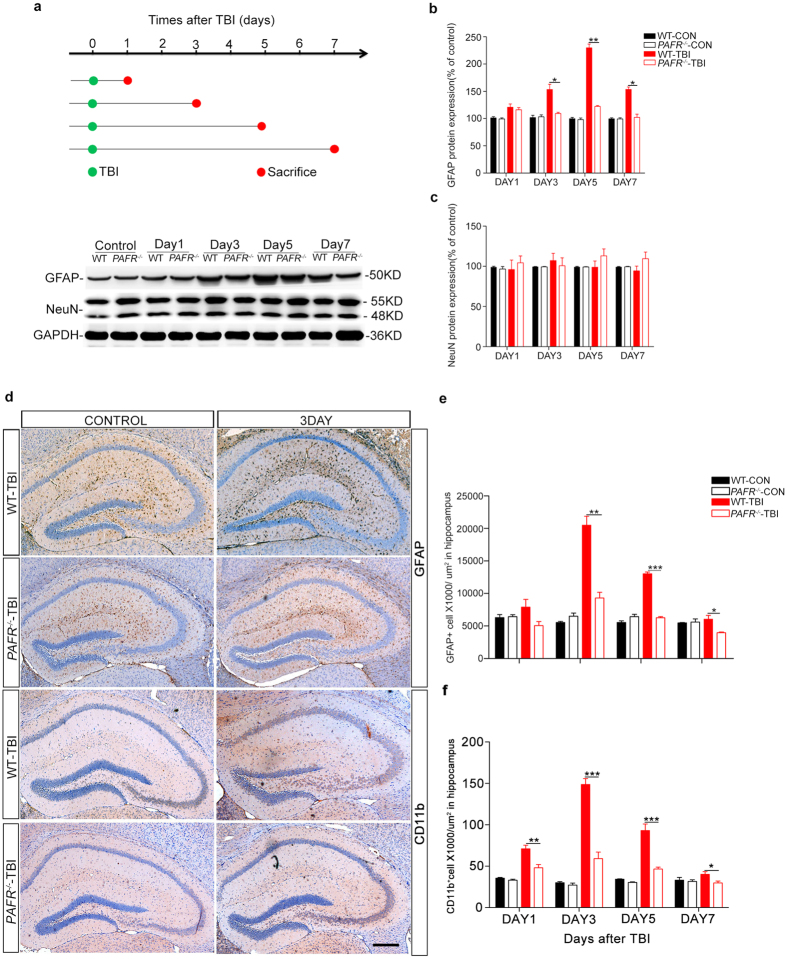
*PAFR*^−/−^ mice have reduced numbers of activated astrocytes and microglia after TBI. (**a**) Flow sheet for the TBI model and sample collections. At days 1, 3, 5, and 7, respectively, mice were sacrificed and brains collected for western blot and immunohistochemistry analyses. Representative images showing GFAP and NeuN proteins expression after TBI in a time-dependent manner. (**b**) GFAP/GAPDH bar graph, representing the percentage ratio of GFAP to GAPDH in *PAFR*^−/−^-TBI and WT-TBI mice at the above time points, respectively. (n = 6) (**c**) NeuN/GAPDH bar graph representing the percentage ratio of NeuN to GAPDH in *PAFR*^−/−^-TBI and WT-TBI mice at the above time points, respectively. (n = 6) (**d**) Representative immunohistochemical images of GFAP-positive astrocytes and CD11b-positive microglia in hippocampal sections after TBI at day 1, 3, 5 and 7, respectively. (**e,f**) GFAP and CD11b-positive cells counts in every μm^2^ in hippocampal sections (n ≥ 6 in the WT-TBI and control group; n ≥ 8 in *PAFR*^−/−^-TBI group). Scale bar, 500 μm. *P < 0.05, **P < 0.01, ***P < 0.001. One-way ANOVA.

PAF has been reported to involve in platelet aggregation and mediate inflammatory responses. We then asked whether ablation of *PAFR* gene can effectively inhibit inflammatory activation after TBI. As the inflammatory response always followed by the activation of astrocytes, we assessed the protein level of GFAP, a marker of astrocytes, in different times after TBI. Interestingly, western immunoblot analysis revealed an evidently increased expression of GFAP in the hippocampus of WT mice after TBI, indicating an inflammatory response arises after TBI. However, protein level of GFAP in *PAFR*^−/−^ mice was significantly lower than WT mice after TBI (Fig. 3a,b). In addition, a time dependent analysis showed unchanged levels of NeuN protein in all experimental groups, which TBI or absence of *PAFR* did not damage neurons of hippocampus (Fig. 3c). Finally, immunohistochemical staining with GFAP and CD11b antibody further confirmed the results (Fig. 3d–f). These results indicate that PAF could mediate inflammatory response after TBI and knock-out *PAFR* can inhibit inflammation-related astrocytes and microglia activation to protect neuron from secondary neuroinflammatory response.

### The expression of inflammatory cytokines in *PAFR* ^−/−^ mice is significantly reduced after TBI

**Figure 4:**
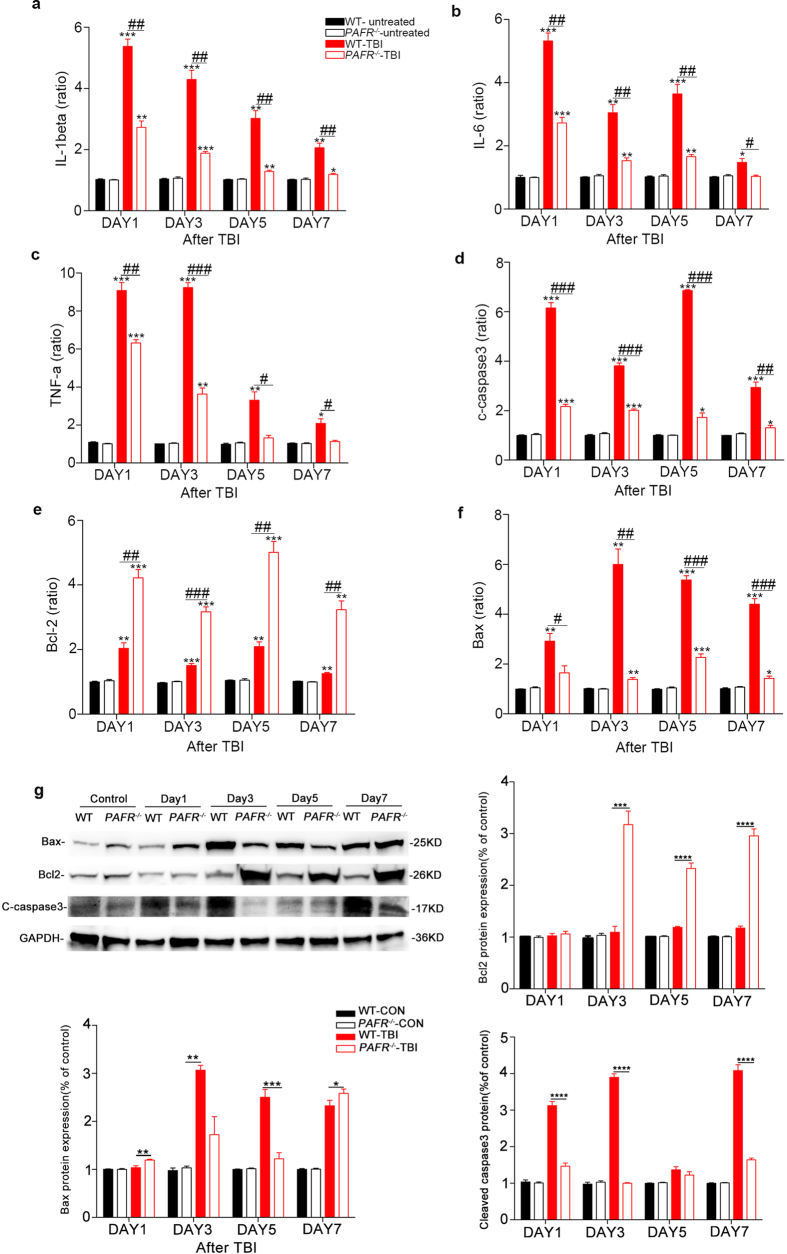
RT-PCR analysis of mRNA expression levels of inflammatory cytokines in *PAFR*^−/−^-TBI group and WT-TBI group at different time points. (**a–c**) IL-1β, IL-6 and TNF-α expression levels. The levels of IL-1β and IL-6 were increased in WT-TBI group 24 h after TBI, while the highest level of TNF-α was found in 72 h after TBI, all of which were significantly higher than that found in the *PAFR*^−/−^-TBI group at similar time points. (**d–f**) The expression levels of Cleaved caspase-3, bcl-2 and bax. Cleaved caspase-3 mRNA levels in *PAFR*^−/−^-TBI mice were significantly lower than in WT-TBI mice. (**g**) Protein expression of apoptosis and anti-apoptosis molecules in hippocampus of wild type and *PAFR*^−/−^ mice. Bcl2 expression is up-regulated, while cleaved caspase-3 and bax are down-regulated in *PAFR*^−/−^-TBI mice. *P < 0.05, **P < 0.01, ***P < 0.001, ****P < 0.0001, for experimental groups compared with control group. ^#^P < 0.05, ^##^P < 0.01, ^###^P < 0.001, for WT-TBI mice compared to *PAFR*^−/−^-TBI mice. (n ≥ 6) One-way ANOVA.

To investigate how PAF mediates inflammatory response after TBI, we detected the mRNA expression levels of pro-inflammatory cytokines at day1, 3, 5 and 7 after TBI in hippocampal tissue from both *PAFR*^−/−^ and WT mice. The mRNA levels of IL-1β, IL-6 and TNF-α were measured in hippocampal tissue after TBI, with results indicating that expression of IL-1β, IL-6 and TNF-α significantly reduced at day 1,3,5 and 7 in *PAFR*^−/−^-TBI mice compared to WT-TBI mice (Fig. 4a–c). In addition, we also investigated neuronal apoptosis as assessed by the mRNA expression levels and the proteins expression of cleaved caspase-3 and bax/bcl-2. Both mRNA and proteins expression levels of cleaved-caspase3 and bax/bcl-2 in the hippocampus of *PAFR*^−/−^-TBI mice were significantly decreased compared to the WT-TBI group at day 1,3,5 and 7, respectively (Fig. 4d–g). Altogether, these results suggest that TBI could lead to inflammatory response and neuronal apoptosis which were destructive to neuronal functions and this process could be protected by loss of *PAFR*.

### Comparative observation of spine density and postsynaptic density (PSD) between wild type mice and *PAFR* ^−/−^ mice after TBI in hippocampus

**Figure 5:**
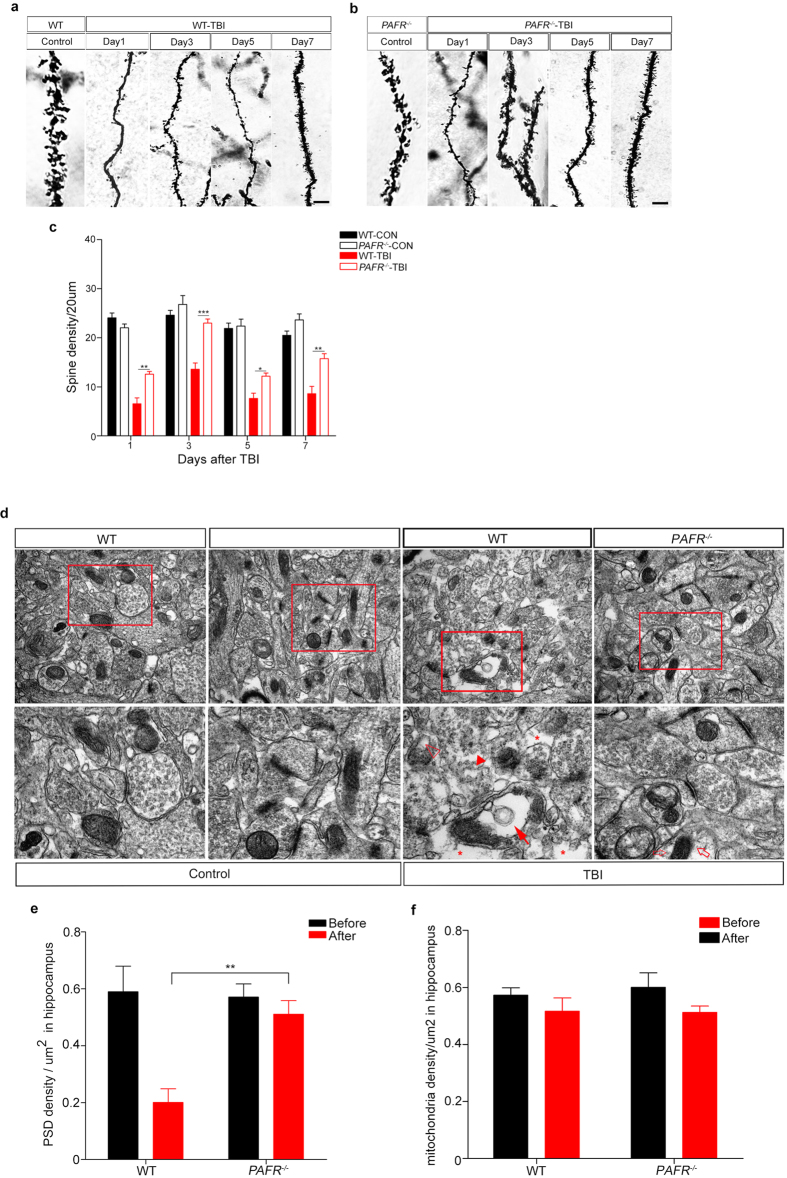
Confocal and transmission electron microscopy of hippocampal dendritic spine and PSD. (**a**) Dendritic spine density at different time points in WT-TBI and in WT-CON mice, respectively. Scale bar = 10 μm. (**b**) Dendritic spine density at different time points in *PAFR*^−/−^-TBI and *PAFR*^−/−^-CON mice, respectively. As shown in the figure, there was no difference of the spine density between the control groups. Scale bar = 10 μm. (**c**) WT-TBI group spine density was significantly reduced compared to *PAFR*^−/−^-TBI group spine density at all time points measured. (**d**) Representative electron microscopy images of hippocampal neuronal PSD from each experimental group, respectively. Controls showed normal PSD. WT-TBI mice exhibited altered mitochondria (full arrows), including swelling and dissolving of the crest, blebs appearing in the peripheral zone (asterisks), evidence of presynaptic vesicle edema (empty arrowheads), as well as fragmentation of the plasma membrane resulting in ill defined PSD boundaries (full arrowheads). There was a significant reduction in alterations to mitochondria and fragmentation of the PSD was less evident in *PAFR*^−/−^-TBI mice. Only a slight cytoplasm dissolved (empty arrows). Scale bar: 500 nm, 200 nm. (**e**-**f**) PSD density and mitochondrial counts statistics per μm^2^ in hippocampal sections. *P < 0.05, **P < 0.01, ***P < 0.001. One-way ANOVA. All the samples were from three independent experiments.

Recent study has reported that dendritic spine density could influence neuronal development, morphology and plasticity, as well as functional consequences on learning and memory formation^22^. We then asked whether TBI may breach dendritic spine density and whether knock out of *PAFR* could rescue this process. As expected, the WT-CON group and *PAFR*^−/−^-CON group showed no differences in dendritic spine density of hippocampus (Fig. 5a,b). However, TBI destroyed the dendritic spine in WT mice and showed a sharply decreased PSD density (Fig. 5a). Interestingly, spine density in *PAFR*^−/−^-TBI mice decrease slightly compared to WT-CON mice or *PAFR*^−/−^-CON mice while far more than WT-TBI group at day 1,3,5 and 7, respectively (Fig. 5a,b and c).

Finally, to see with more detail about the morphological changes after TBI, transmission electron microscopy was used and we found that the mitochondria in hippocampal neurons of both WT-CON group and *PAFR*^−/−^-CON group were rod-like in shape, and exhibitied mitochondrial crest that were uniform in shape with no visible vacuoles. Likewise, the PSD appeared normal and intact. WT-TBI group contained more mitochondria with vacoules and greatly reduced numbers of PSD that frequently showed fragmentation without uniform boundaries (Fig. 5d). PSD were signficantly increased and showed only minor fragmentation in *PAFR*^−/−^-TBI group compared to the WT-TBI group (Fig. 5e). Interestingly, mitochondria in hippocampal neurons of *PAFR*^−/−^-TBI group appeared relatively normal but mitochondria number statistics have no difference in both WT-TBI group and *PAFR*^−/−^-TBI group (Fig. 5f). These data suggest that *PAFR* KO conveys protective effects on the dendritic spine morphology and density of hippocampal neurons after TBI.

## Discussion

The present study revealed that PAF and its associated receptor strongly mediates the inflammatory response after TBI. We found that ablation of the PAFR receptor significantly reduced inflammation and promoted protection of hippocampal neurons after TBI.

We found that *PAFR*^−/−^ mice exhibited greater tolerance when subjected to TBI and showed reduced levels of neuroinflammation compared to their WT littermates. Moreover, these mice showed enhanced ability in the MWM, further supporting the conclusion from previous drug studies that inhibition of the PAFR can protect learning and memory ability and significantly attenuate PAF-induced neurotoxicity after TBI^23^,^24^. Furthermore, we evaluated the working memory function of mice after TBI using consecutive water maze measurements with a hidden platform paradigm, and we found that the escape latency was significantly higher in the WT-TBI group and *PAFR*^−/−^-TBI group compared with the WT-CON and *PAFR*^−/−^-CON groups, respectively, and the difference was nearly 30 s at 6 days after injury. However, Gao^25^ found that the GCPII^−/−^-TBI group had significantly shorter latency times while walking the beam compared to the WT-TBI group on days 14, 21, and 28 post injury, and there were no differences between the early escape latency times. We postulate that the distinct results between the above study and the current one are due to the knockout of different genes as well as resulting differences which influenced cognitive function after TBI validation.

After TBI injuries often associated to the peripheral tissue can exacerbate neuroinflammation^26^. Indeed, human gene polymorphism analyses indicate that various inflammatory factors are increased after TBI^27^. Inflammation can occur due to activation of inflammatory cytokines, resulting in secondary damage to the brain which in turn leads to a decline in learning ability as well as reduced memory and cognitive function^28^. In addition loss of *PAFR* may ameliorate the loss of spatial learning ability and reduced memory and cognitive function after TBI. A variety of potent lipid mediated receptor inhibitors, including BN52021 and WEB2086, have been characterized^20^,^29^ and shown to have therapeutic potential in a number of neurological disorders, including models of ischemia-reperfusion injury^30^, neuroinflammatory disease^31^, neurotoxic injury^32^. PAFR inhibitors display neuroprotective properties after TBI^33^,^34^,^35^,^36^ and these compounds might exert their effects via an inhibitory mechanism involving the interaction between PAF and PAFR^37^,^38^. Our studies have demonstrated that PAFR inhibitors caused a reduction in PAF release resulting in a down regualtion of TBI induced inflammatory cytokines^39^,^40^,^41^ in animal models of brain injury. To further verify the hypothesis that inhibition of PAFR augments an endogenous protective mechanism after TBI, we used the homologous recombination method to generate *PAFR*^−/−^ mice. Mice lacking the *PAFR* gene developed normally with no obvious differences from WT mice. In our study we demonstrated a prior study that investigated the efficacy of *PAFR* inhibitionin the hippocampus after TBI^42^. Results from the current study indicated that ablation of *PAFR* reduced the inflammatory response induced by TBI. In addition, loss of *PAFR* reduced astrocyte infiltration and activation in the hippocampus after TBI, which may have been responsible for the reduced expression of inflammatory cytokines found in the study. These results also further lend support to the evidence of intracerebral inflammatory mechanisms in patients after TBI.

Most TBI patients exhibit disabilities associated with cognitive impairments^43^ that include attention-deficit disorder, loss of executive functions and working memory, all of which can last for months or years after TBI^44^. Results from the current study indicated that PAF plays an important role in the inflammatory response after TBI, and knockout of *PAFR* can effectively control this response, leading to improved cognitive function and accelerate injury recovery after TBI. Learning and memory are known to be affected by synaptic function^45^,^46^ and in particular, dendritic spine formation^47^, as it has been shown that shrinkage and/or loss of spines are associated with memory loss^48^. Our results regarding increased PSD and overall structural integrity in *PAFR*^−/−^ mice after TBI are in agreement and suggest a relationship between hippocampal synapse ultrastructure and learning/memory. Ablation of *PAFR* gene significantly reduced the levels of expression of inflammatory cytokines including IL-6, IL-1β and TNF-α, as well as apoptosis related markers cleaved caspase-3 and bax/bcl-2, and also reduced the numbers of activated astrocytes in the hippocampus after TBI. Although these findings indicate a role for knockout of *PAFR* in the suppression of these factors, the exact mechanisms involved are yet to be elucidated.

## Methods

### Animals

Adult wild-type male C57/BL6 and *PAFR* KO mice (8–10 weeks), weighing approximately 25 ± 3 g, were generated and housed in The Southern Model Biological Technology Development Co. (Shanghai, China). The knockout strategy was as follows; exon 2, which encodes for the *PAFR*, was replaced with the PGK - neo - pA following homologous recombination in embryonic stem cells and the neo and TK cassettes were used for positive and negative selections. Animals were genotyped by PCR with forward primer GGACGCCAACATCCAAACT and reverse primer GTAGAATCCAGTCGCCCTCG for wild type (404 bp), and forward primer GGACGCCAACATCCAAACT and reverse primer GAAAGCGAAGGAGCAAAGC for mutant (556 bp). Animals were housed and bred in a pathogen free feeding center under controlled conditions (temperature 24 ± 1 °C and humidity 70 ± 5%) and a 12:12 h light: dark cycle. Animals were handled daily throughout the experimental session in order to acclimate them with the research personnel thereby reducing stress. All experiments involving mice were carried out in accordance with the US National Institutes of Health Guide for the Care and Use of Animals under an Institutional Animal Care and Use Committee approved protocol and Association for Assessment and Accreditation of Laboratory Animal Care approved Facility at the Shanghai Jiao Tong University School of Medicine.

### Reagents

Antibodies were purchased from Cell Signaling Technology (Beverly, MA, USA), including: monoclonal anti-glial fibrillary acidic protein (GFAP) antibody, monoclonal anti-cleaved caspase-3, monoclonal anti-bcl2 antibody, monoclonal anti-bax antibody, monoclonal anti-NeuN antibody and monoclonal anti-GAPDH antibody. Monoclonal anti-Tau from Millipore (Billerica, MA, USA), monoclonal anti-Tuj1 from Beyotime (Shanghai, China), monoclonal anti-Calbindin from SWANT (Bellinzona, Ticino, Switzerland). The RT-PCR and kits were purchased from TaKaRa (Shiga, Japan). Experimental materials including: MicroAmp^®^ fast 8-tube strip and optical 8-cap strip, MicroAmp^®^ fast optical 96 well reaction plate with barcode, MicroAmp^®^ 96-well optical adhesive film were from Applied Biosystems^TM^, Life Technologies Co. (Grand Island, NY, USA). Nucleic acid electrophoresis agarose and trizol were purchased from Invitrogen^TM^, Life Technologies Co.(Grand Island, NY, USA).

### TBI Procedure

All test were conducted according to previous study^25^. Briefly, littermate mice were divided into four groups in this experiment, including WT-CON group, *PAFR*^−/−^-CON group, WT-TBI group and *PAFR*^−/−^-TBI group, respectively. An accurate cortical injury model was established using the PCI3000 Precision Cortical Impactor (Hatteras Instruments Inc., Cary, NC, USA), which consists of ahead holder, stereotaxic frame, a pressure transducer, and a skull bore device. Mice undergoing TBI were anesthetized with 1% pentobarbital sodium for induction. Anesthesia was maintained with 2% isoflurane for the duration of surgical preparation in the first place. Then the head was fixed in the stereotaxic instrument to prevent head movement at the moment of impact, and a 3 mm diameter craniotomy centered at 2 mm posterior to bregma and 2 mm lateral to the midline over the right hemisphere was performed. The Pin-Point ^TM^ (Model PCI3000) controlled cortical impact device was attached to a 3 mm rounded metal tip that was angled (20–30°) to have a vertical direction to the skull surface. The device was used to deliver a middle degree injury with an impact velocity of 3.0 m/s, at a depth of 1.0 mm, and a duration of 180 ms. After impact, the wound was gently sutured, and the mouse was removed from the stereotaxic holder and the core body temperature was monitored by a rectal thermometer and maintained at 37 °C on a heating pad.

### Western Immunoblots

For western immunoblot, hippocampus were dissected and lysed in ice-cold radio immunoprecipitation assay buffer supplemented with a protease and phosphatase inhibitor cocktail solution (Millipore, Billerica, MA, USA). Protein concentrations were determined using the bicinchoninic acid assay (Beyotime, Shanghai, China)^49^ after TBI 1,3,5 and 7 days respectively. 30 μg protein of each sample was loaded on a 12% SDS-PAGE gel and electro-transferred onto a nitrocellulose membrane (Millipore). The membranes were blocked with 5% degreased milk and incubated with primary antibodies against GFAP (1:1000 dilution), NeuN (1:1000 dilution), cleaved caspase-3 (1:300), bcl2 (1:300) and bax (1:300) with gentle agitation at 4 °C overnight. The membranes were then incubated with goat anti-rabbit or goat anti-mouse secondary antibodies for 1 h at room temperature and immunoreactive protein bands were detected and visualized with the Image Quant LAS 4000 Mini (GE Healthcare Bio-Sciences, USA). Densitometric measurements of band intensities were analyzed using Adobe Photoshop CC 14.0 software.

### Morris Water Maze (MWM)

Adult mice (8–10 weeks) after one day for TBI were measured using the MWM as previously described^50^. Briefly, Morris water maze test begins in the first day after TBI, control and experimental groups were trained for place and probe trials in a tank 116 cm in diameter and 50 cm in depth, with a hidden 8 cm diameter platform 1.0 cm below the surface of water fixed in the target quadrant. The place trials were conducted five times for five successive days and the probe trials were performed on the sixth day. For each place trial, mice were randomly placed into four quadrants to swim freely for a maximum of 60 s. If the platform was not found, the mice were guided to the platform and left for 10 s for memory purposes. The latency time to find the platform, run across the target quadrant, time spent in the target quadrant, and distance in the target quadrant were all recorded. For probe trials, the platform was removed and the mice were introduced to the quadrant opposite to where the platform was located and allowed to swim for 60 s. Spatial memory ability was measured by the time spent in the target quadrant compared to the rest quadrants.

### Open Field Test (OFT)

Open-field apparatus consisted of four chambers (60 × 60 × 30 cm) as previously described^51^. Each mouse was placed in a corner of the open field under low light conditions. Lighting consisted of four 100 watt incandescent light bulb placed in the four directions of the testing room 3 m from testing arena. Each trial lasted for 20 min with 1 trial per mouse. Between sessions, the chamber was rinsed with 75% ethyl alcohol. Time in center area, distance traveled in center area, total distance and grooming time in the test were used as locomotory ability, anxiety indexes and living habits. The results were analyzed using EthoVision software (Noldus Information Technology, Leesburg, VA).

### Elevated Plus Maze (EPM)

All tests were conducted according to a previous study^52^. Mice were given 1 h to habituate before EPM tests were conducted. All apparatuses and testing chambers were cleaned with 75% ethyl alcohol wipes between animals. The EPM apparatus was made of dark grey plastic and consisted of two open arms (30 7 0.25 cm) opposed to two enclosed arms (30 7 15 cm) elevated 60 cm from the floor. Animals were placed in the central area of the apparatus with their head facing an enclosed arm (test duration: 5 min). The test was performed in a sound-attenuated and temperature-controlled (23 ± 1 °C) room illuminated by one 40-W fluorescent bulb placed 3 m above the apparatus. Digitized video recordings with EthoVision software (Noldus Information Technology, Leesburg, VA) were used for behavioural analysis. The percentage of time spent in open arms and the percentage of open-arm entries were used as innate fear indexes.

### Immunofluorescence Staining

The immunofluorescence was performed as we described previously^52^, with minor modification. Briefly, brain slides containing the whole hippocampus were washed three times for 10 min in 0.1 M PBS, followed by incubation for 1 hour in blocking solution containing 10% normal donkey serum. After blocking, the slides were incubated overnight at 4 °C in the primary antibodies. Then the sections were incubated with Alexa Fluor 488 donkey anti-rabbit and 555 donkey anti-mouse (Invitrogen^TM^, Life Technologies Co., Grand Island, NY, USA) for 2 h at room temperature. Staining patterns of the immunofluorescence were examined using a confocal laser-scanning microscope (Flouview FV 1000).

### Primary Culture

Hippocampal neurons were cultured following methods in the previous study^53^. Briefly, wild type mice and *PAFR*^−/−^ mutants at P1-P3 were dissected, hippocampal neurons were digested with 1 mg/ml papin and 50 μl DNAase for 30 min at 37 °C and terminated by adding 3 mL Neurobasal-A medium containing 2% B27, 10% fetal bovine serum and 0.5 mM L-glutamine. The cell seeded onto multi-well culture plate precoated with 0.1 mg/mL poly-D-lysine. Following culture at 37 °C in a humidified atmosphere of 5% CO_2_. Later, half of medium was replaced every 3 days and 5 μmol/L PAF added to the medium before the cells harvest 24 hours. Cells were used for experiments at 7–9 days *in vitro* (DIV).

### RT-PCR

Hippocampus tissue was dissected after TBI 1,3,5 and 7 days respectively and RNA was isolated for RT-PCR, using trizol reagent (Invitrogen ^TM^) according to the manufacturer’s instructions, and purity was measured by absorbance at 260 and 280 nm. Total RNA samples (2 μg) were reverse-transcribed with the Prime Script RT Reagent Kit (Perfect Real Time, TaKaRa Biotechnology) according the manufacturer’s protocol and mixed with SYBR Select Master Mix (Life Technologies Co.). The mRNA levels of IL-6, IL-1β, TNF-α, cleaved caspase-3, and bax/bcl-2 were quantitatively measured using the Applied Biosystems step-one plus Real-Time PCR (Life Technologies Co.). *GAPDH* was used as an internal control for all samples. The sequences of the forward and reverse primers are listed in Table 1. The RT-PCR program has been previously desribed^54^. The threshold cycle (Ct) was calculated using the second-derivative maximum method and data were calculated via the delta−delta method. The assays were performed three times using triplicate wells. The final values are expressed as the ratio versus the control group.

### Immunohistochemistry

The immunohistochemistry staining protocol was established as previously described^55^. Briefly, mice were anesthetized with 1% pentobarbital sodium and perfused with ice cold 0.1 M PBS (PH = 7.4) followed with 4% PFA in 0.1 M PBS at the different time points after TBI. The brains were quickly removed and kept in 4% PFA overnight at 4 °C. After embedding in paraffin, coronal sections (6 um) were sliced on a cryostat. Sections were incubated in primary antibody rabbit anti-GFAP (1:200, Cell Signaling Technology) and mouse anti-CD11b (1:200, Abcam, cambridge, USA) in normal goat serum for 2 h at 37 °C. Sections containing the hippocampus were imaged using a microscope (Leica DM4000B, Leica Microsystems, Germany) at 5 times (5x) magnification. The numbers of positive cells in an area of per um^2^ were counted (n ≥ 6/group). Twelve sections in each brain were collected and analyzed.

### Golgi Staining

Brain tissue were harvested at the same time points with western blot. After brain removal, hemispheres were immersed in 10 ml modified Golgi-Cox staining solution (courtesy of D. Jing and F. Lee, Cornell University, Ithaca, NY, USA) for 10–14d at room temperature in the dark, as previously described^56^. Sections (100 μm) were mounted onto slides coated with 0.3% gelatin in double distilled (dd) H_2_O and dried overnight in the dark. Slides were immersed in ddH_2_O three times for 10 min with gentle shaking and then transferred to a developing solution for 6 min. Slides were then rinsed three times for 10 min in ddH_2_O, dehydrated through graded ethanol solutions (50%, 75%, 95%,100%), immersed in xylene two times for 5 min and then quickly cover slipped using DPX mounting medium (Sigma, USA). Neurons were traced at 100×, and all subsequent analyses were done using Image-Pro Plus (Version 6.0.0). Analysis was performed by quantifying the number of dendritic spines on a single hippocampal neuron. All counting was performed in a blind manner.

### Transmission Electron Microscopy (TEM)

TEM (Model JEM-1200 EX, JEOL, Japan) methods were used as previously described^57^ in order to characterize the morphology of the synaptic connections as well as the ultrastructure of neurons. Hippocampus tissue were harvested at the third day after TBI which acute phase of inflammation and edema appeared. Hippocampus tissue was cut into 1 cm ×1 cm sections and embedded in paraffin. Two-three days after seeding, the tissue wasfixed with 2.5% glutaraldehyde (pH 7.4) for 24 h, followed by treatment with 1% osmium tetroxide for 4 h. The tissue was dehydrated in a graded series of ethanol solutions (30%, 50%, 70%) for 10 min, critically point dried and coated with gold. Ultrathin sections of hippocampal tissue were stained with lead citrate and uranyl acetate and observed via transmission electron microscopy (Model JEM-1200 EX, JEOL). The quantification of spine density per 20 μm from 15 visual fields of three animals per genotype at a magnification of 33,000× were shown.

### Statistical Analysis

The differences of index were evaluated by mean ± SEM in all experiments. Statistical differences were determined by Student’s t-test for two-group comparisons or analysis of variance (ANOVA) followed by Dunnett’s test for multiple comparisons among more than two groups.

## Additional Information

**How to cite this article**: Yin, X.-J. *et al*. Loss of PAFR prevents neuroinflammation and brain dysfunction after traumatic brain injury. *Sci. Rep.* **7**, 40614; doi: 10.1038/srep40614 (2017).

**Publisher's note:** Springer Nature remains neutral with regard to jurisdictional claims in published maps and institutional affiliations.

## Figures and Tables

**Table 1:** Primer sequences.

Primer	Sequence
GAPDH-F	5′ AAA TGG TGA AGG TCG GTG TG
GAPDH-R	5′ AGG TCA ATG AAG GGG TCG TT
Caspase3-F	5′ TCG CAG CAT TTC TCC TAA G
Caspase3-R	5′ CAA CAA AGC CAG TCT AAA CA
IL-1beta-F	5′ GTA CAA GGA GAA CCA AGC AA
IL-1beta-R	5′ CCG TCT TTC ATT ACA CAG GA
IL-6-F	5′ CAA ATG CTC TCC TAA CAG AT
IL-6-R	5′ TGT CCA CAA ACT GAT ATG CT
TNF-a-F	5′ ACT CTG ACC CCT TTA CTC TG
TNF-a-R	5′ GAG CCA TAA TCC CCT TTC TA
Bcl2-F	5′ CGT GTA ACT TGT AGC GGA TA
Bcl2-R	5′ ATG CTG AAA GGT GAA GAG G
Bax-F	5′ GGA GAT GAA CTG GAC AGC AA
Bax-R	5′ CAA AGT AGA AGA GGG CAA CC

Table for the RT-PCR primers and the sequences of primer upstream and downstream.
